# Long-Term Impact of Cyclosporin Reduction with MMF Treatment in Chronic Allograft Dysfunction: REFERENECE Study 3-Year Follow Up

**DOI:** 10.1155/2010/402750

**Published:** 2010-07-28

**Authors:** L. Frimat, E. Cassuto-Viguier, F. Provôt, L. Rostaing, B. Charpentier, K. Akposso, M. C. Moal, P. Lang, D. Glotz, S. Caillard, D. Ducloux, C. Pouteil-Noble, S. Girardot-Seguin, M. Kessler

**Affiliations:** ^1^Service de Néphrologie/Transplantation, CHU de Nancy, rue du Morvan, 54511 Vandoeuvre-Les-Nancy, France; ^2^Service de Néphrologie, CHU Pasteur, 30 avenue de la Voie Romaine, BP 69, 06000 Nice, France; ^3^Service de Néphrologie, CHRU Albert-Calmette, boulevard Professor Jules Leclerc, 59037 Lille Cedex, France; ^4^Service de Néphrologie, CHU Rangueil, avenue Jean Poulhes, 31403 Toulouse, France; ^5^Service de Néphrologie, CHU Bicêtre, 78, rue du Général Leclerc, 94270 Le Kremlin Bicètre, France; ^6^Service de Néphrologie, Polyclinique de Blois, 1 rue Robert Debré, 41 260 La Chaussée-Saint-Victor, France; ^7^Service de Transplantation, Hôpital de la Cavale Blanche, boulevard Tanguy Prigent, 29609 Brest Cedex, France; ^8^Service de Néphrologie et Transplantation, Hôpital Henri Mondor, 51 avenue de Lattre de Tassigny, 94010 Creteil, France; ^9^Service de Néphrologie et Transplantation, Hôpital Saint-Louis, 75010 Paris, France; ^10^Service de Néphrologie, CHU, 1, place de l'Hôpital, 67009 Strasbourg Cedex, France; ^11^Service de Néphrologie, Hôpital Saint-Jacques, Place Saint-Jacques, 25000 Besançon, France; ^12^Service de Néphrologie et Transplantation, Hospices Civils Lyon Sud, Chemin du Grand Revoyet, 69495 Lyon Cedex, France; ^13^Roche Pharma, 52, boulevard du Parc, 92521 Neuilly-sur-Seine Cedex, France

## Abstract

Calcineurin inhibitor (CNI) toxicity contributes to chronic allograft nephropathy (CAN). In the 2-year, randomized, study, we showed that 50% cyclosporin (CsA) reduction in combination with mycophenolate mofetil (MMF) treatment improves kidney function without increasing the risk for graft rejection/loss. To investigate the long-term effect of this regimen, we conducted a follow up study in 70 kidney transplant patients until 5 years after REFERENCE initiation. The improvement of kidney function was confirmed in the MMF group but not in the control group (CsA group). Four graft losses occurred, 2 in each group (graft survival in the MMF group 95.8% and 90.9% in control group). One death occurred in the control group. There was no statistically significant difference in the occurrence of serious adverse events or acute graft rejections. A limitation is the weak proportion of patient still remaining within the control group. On the other hand, REFERENCE focuses on the CsA regimen while opinions about the tacrolimus ones are still debated. In conclusion, CsA reduction in the presence of MMF treatment seems to maintain kidney function and is well tolerated in the long term.

## 1. Introduction

Renal transplantations permit quality of life improvement for patients presenting with chronic renal insufficiency, in addition to increasing their life expectancy. Recent years were marked by a reduction of acute rejection episodes and improved graft survival in the short term as a result of the use of calcineurin inhibitors (CNIs), without progress in long-term graft survival [1]. This observation is attributable to the nephrotoxic effect of CNI [2], the long-term use of which is implicated in the development of chronic allograft lesions and suggested to contribute to chronic allograft nephropathy (CAN). 

It is thus considered primordial to avoid CNI toxicity while at the same time minimizing the risk of renal dysfunction and graft loss. Consequently, a number of studies were conducted with reduction, withdrawal, or avoidance of CNI, in particular of cyclosporin reviewed in [3, 4]. The first attempts of CNI withdrawal were associated with a significant increase in acute rejection incidents, thus rendering the exact appraisal of the benefit/risk ratio of such a regimen difficult [5–7]. The advent of new immunosuppressive agents such as mycophenolate mofetil (MMF) revived the interest in alternative immunosuppressive treatment strategies.

MMF is the ester prodrug of mycophenolic acid (MPA), which selectively and reversibly inhibits the rate-limiting enzyme in the de novo biosynthesis of guanosine nucleotides [8]. MMF reduces the risk of acute allograft rejection incidents [9], without nephrotoxic side effects [10], which is suggestive of an ideal candidate for long-term CsA reduction treatment strategies. Short-term studies have proven the positive impact of CsA dose reduction with concomitant MMF administration on renal graft function [11]. In a recent publication concerning the DICAM study, a prospective randomized trial confirms these results [12]. However, the study of long-term consequences of this approach is important, given the possibility that the observed short-term improvements might be the outcome of the elimination of the functional CNI nephrotoxicity of vascular origin, and that lesions in relation to reduced immunosuppression could eventually result in graft loss. Therefore, to address the question whether renal function improvement in patients with chronic allograft dysfunction observed in the initial two-year REFERENCES study [13] would be maintained in the long term, we conducted a follow up study for 5 years after study initiation.

## 2. Materials and Methods

### 2.1. Study Design and Study Patients

The REFERENCE study (Renal function evaluation after half dose reduction of Neoral in combination with CellCept in renal transplant patients with altered renal function) was an open, randomized, controlled, multicenter, prospective study, conducted in 15 centers in France between March 2000 and February 2007. The study was conducted in accordance with the Declaration of Helsinki. All patients gave their written informed consent before entering the study, after the protocol and the informed consent form were approved by an Independent Ethics Committee (IEC of Lorraine, France).

Initially, a study duration of 96 weeks was planned, which was extended to five years with the approval of the IEC. Study design and inclusion criteria for the initial study were previously described in detail in [13]. Briefly, eligible patients were between 18–65 years old, had received a first or second renal graft from a deceased or living donor one to ten years prior to the study, and were receiving a CsA-based immunosuppressive treatment for at least three months. Patients presented with CAD which was defined by altered renal function as indicated by a serum creatinine level between 1.7 and 3.4 mg/dL. Eligible patients were randomly assigned to one of two treatment arms. Patients in the MMF group received a dose of 2 g MMF per day with half the dose of CsA compared to the initial dose. Azathioprine treatment was to be stopped before the introduction of MMF. In the control group, patients received CsA according to the center's practice, with a minimal detectable target through level of 100 ng/mL. In both treatment arms corticosteroids were prescribed following the practice of the center. 

**Figure 1:**
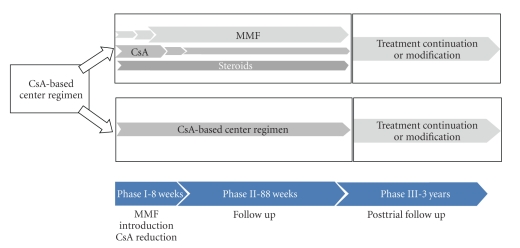
Study design.

After completion of the initial study (96 weeks), patients could choose to participate in the three-year follow up phase, thus leading to a total study duration of five years. Patients had to give their written informed consent for study continuation. The details of the study design are presented in Figure 1. The follow up phase required six semiannual follow up visits which included a clinical and a laboratory exam. The protocol did not define any treatment for this period. Patients either continued with the treatment they received during the initial study phase, or a change of the immunosuppressor treatment was implemented at the discretion of the investigator. The study populations were thus defined as follows. Randomization population: *MMF group*–patients randomized to receive a 50% reduction of CsA. *Control group*—patients randomized to receive the usual CsA dose. On-treatment population: *Group I*—patients who received a treatment with a mycophenolic acid derivative (MMF or mycophenolate sodium, MPS) at the end of the follow up phase. *Group II*—patients without such a treatment at the end of the follow up phase. 

### 2.2. Primary and Secondary Endpoints

The study objective was to determine if administration of MMF in combination with CsA reduction by 50% leads to improvement of allograft function on the long term. The primary efficacy endpoint during the three-year follow up phase was the evolution of allograft function as evaluated by 1/SeCr (inverse of serum creatinine) between week 96 and the end of the three-year follow up phase. 

As secondary endpoints to assess allograft function creatinine clearance (calculated with the Cockcroft's formula) and proteinuria were analyzed. Additional analyses included graft and patient survival, occurrence of acute graft rejection, recurrence of initial nephropathy, adverse events, and laboratory parameters.

### 2.3. Statistical Analysis

No statistical hypothesis was formulated for the three-year follow up phase. The analysis populations were the randomization population and the on-treatment population as described above. This comprised all randomized patients who completed the initial study phase and gave their consent to participate in the three-year follow up. Results were expressed as mean ± standard deviation (SD) for quantitative variables and absolute and relative frequencies for qualitative variables. Continuous variables were compared using analysis of variance while categorical variables were analyzed with a chi-square test or the Fisher's exact test. Statistical analyses were performed with a two-sided test with a significance level of 5% using SAS software (version 8.2, SAS Institute, Inc., Cary, NC, USA).

### 2.4. Role of Funding Source

The study sponsor, Roche (Neuilly sur Seine, France), chose the participating centers, funded the creation of the centralized database and the study monitoring, and employed an independent company to conduct the statistical analysis and to participate in the writing of the paper.

## 3. Results

### 3.1. Analysis Population

**Figure 2:**
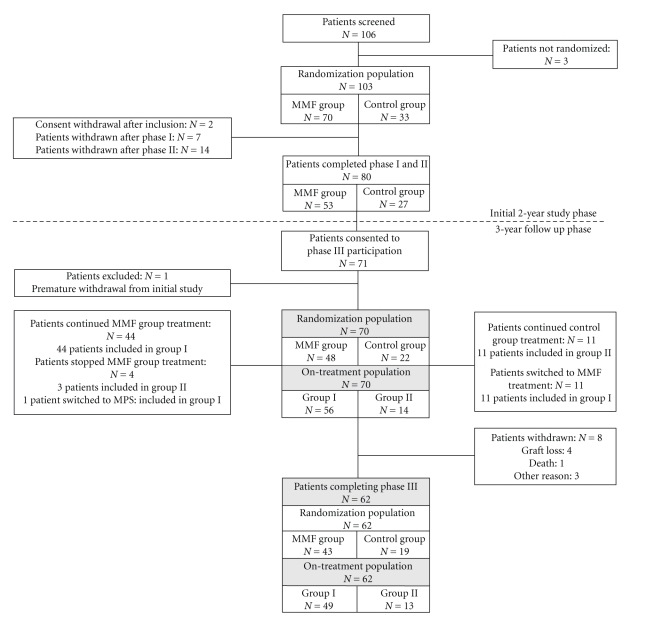
Study flow chart.

Among the 106 patients who were enrolled in the initial study, 103 were randomized, 80 completed it, and 71 gave their informed consent to continue in the three-year follow up phase (Figure 2). One of these patients was excluded from the follow up phase due to premature withdrawal from the initial study. Baseline characteristics and demographics of patients included in the study were previously described [13]. Among the 70 patients who were included in the three-year follow up, 48 were part of the MMF group, and 22 belonged to the control group. For analysis five years after study initiation patients were analyzed according to whether or not they received a mycophenolic acid derivative at the last visit. A total of 15 patients changed treatment during the follow up phase. Three patients from the MMF group stopped MMF treatment and were therefore included in group II, while one patient switched from MMF to MPS and was accounted for in group I. Eleven patients from the control group were treated with MMF and were therefore included in group I. Consequently, group I consisted of 56 patients who were treated with MMF (55 patients) or MPS (1 patient) at the last visit or last available visit for premature withdrawals, and group II included 14 patients who did not receive MMF or MPS treatment at the end of the follow up phase. A total of eight patients (11.4%) withdrew prematurely from the three-year follow up: five patients in the MMF group and three patients in the control group. See Table 1 and Figure 2 for details.

### 3.2. Immunosuppressive Treatment

During the follow up phase patients either maintained or modified the immunosuppressive regimen they were assigned to in the initial study phase. 

Concerning MMF treatment (Table 2(a)) in the randomization population, the mean daily dose gradually decreased from month 30 to month 60. This is mainly due to the decrease of the proportion of patients taking exactly 2 g/day in the MMF group from 75% to 60.5%. In the control group, two patients (9%) received MMF at month 30 and nine (47.4%) at month 60. Unlike previously, in the on-treatment population the mean daily MMF dose and the proportion of patients taking 2 g/day increased in group I, from month 30 to month 60. In group II, there were still three patients treated with MMF at month 48, but none thereafter.

Regarding CsA treatment (Table 2(b)), for the randomization population the mean daily CsA dose in the MMF group remained stable from month 30 to month 60. At the same time the mean daily CsA dose in the control group progressively decreased. For the on-treatment population, the mean daily CsA doses between month 30 and month 60 in group I were comparable to those in the MMF group. The values in group II and control group were also comparable. However, the values at month 48 in group II exceeded 200 mg/day.

### 3.3. Renal Function

**Figure 3:**
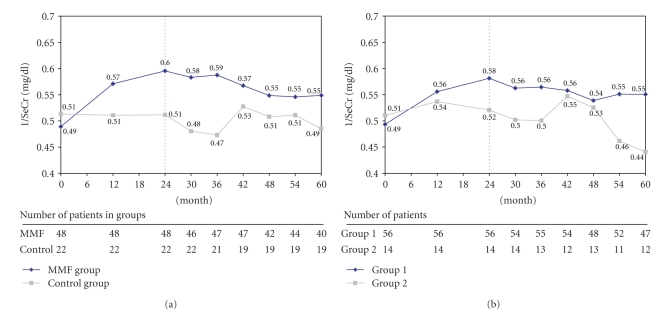
(a) Evolution of inverse of creatinine (1/SeCr) over time in the randomization population (MMF group versus control group). (b) Evolution of inverse of creatinine (1/SeCr) over time in the on-treatment population (group I versus group II). The vertical, dotted line separates initial study phase and follow up phase.

The evolution of renal function as evaluated by the evolution of 1/SeCr in the randomization population is shown in Figure 3(a). In the MMF group, a positive slope of this parameter was observed during the 24 months of the initial study phase. In the course of the follow up phase, the 1/SeCr value, which was 0.49 mg/dl ± 0.08 at baseline and 0.60 mg/dl ± 0.13 at month 24, gradually diminished to reach 0.55 mg/dl ± 0.16 at month 60. It was still significantly higher compared to baseline (*P* = .018) despite a reduction between month 24 and month 60. In the control group, 1/SeCr values remained relatively stable throughout the follow up phase, with 0.51 mg/dl ± 0.08 at baseline, 0.51 mg/dl ± 0.10 at M24, and 0.49 mg/dl ± 0.14 at M60. A transient increase was, however, noted at month 42 (0.53 mg/dl ± 0.10), which was possibly influenced by the withdrawal of two patients due to renal graft loss in this group. No statistically significant change was observed in the control group between baseline and month 60. 1/SeCr level changes between baseline and month 60 were statistically different between the two groups (*P* = .025, 0.06 mg/dl ± 0.15 in the MMF group and −0.03 mg/dl ± 0.11 in the control group), but no statistically significant difference of the 1/SeCr value at month 60 was observed between the two groups (*P* = .134). The evolution of 1/SeCr in group I of the on-treatment population was comparable to the MMF group, resulting in a statistically significant difference between the two groups at month 60 (*P* = .024, Figure 3(b)). In group I, 1/SeCr levels were significantly higher at month 60 compared to baseline (*P* = .008). No such change was observed in group II. The changes between baseline and month 60 were statistically different between the two groups (*P* = .008, 0.05 mg/dl ± 0.13 in group I and −0.06 mg/dl ± 0.13 in group II). 

**Figure 4:**
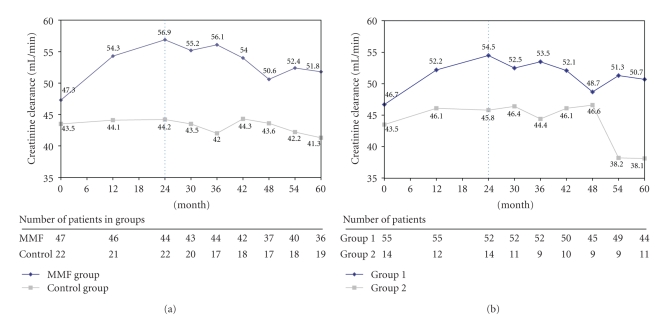
(a) Evolution of creatinine clearance over time in the randomization population (MMF group versus control group). (b) Evolution of creatinine clearance over time in the on-treatment population (group I versus group II). The vertical, dotted line separates initial study phase and follow up phase.

Creatinine clearance as presented in Figure 4(a) increased in the MMF group during the initial study period (47.3 mL/min ± 11.4 at baseline and 56.9 mL/min ± 16.7 at month 24) and gradually diminished during the post-trial period to 51.8 mL/min ± 20.2 at month 60. In the control group this parameter remained stable during the initial study phase (43.5 mL/min ± 12.0 at baseline and 44.2 mL/min ± 14.6 at month 24) and slightly decreased during the post-trial phase to 41.3 mL/min ± 18.9 at month 60. Analysis of group I showed a result similar to the MMF group (Figure 4(b)), while creatinine clearance in group II abruptly fell at month 54 and was 38.1 mL/min ± 22.1 at month 60 compared to 45.8 mL/min ± 16.4 at month 24. The differences observed between MMF and control group and group I and group II at the end of the study were not statistically significant (*P* = .066). Creatinine clearance changes between baseline and month 60 were not significantly different in any of the four groups nor were the changes for the same time period between the MMF group and the control group, and group I and group II. 

### 3.4. Secondary Endpoints

#### 3.4.1. Graft and Patient Survival

A single death was reported during the three year follow up phase. This concerned a patient from the control group who died from a metastatic non-small-cell lung cancer (Table 1). A total of four graft losses occurred, two each in the MMF (graft survival 95.8%) and in the CsA group (graft survival 90.9%) with no statistically significant difference between the two groups (*P* = .585).

#### 3.4.2. Renal Dysfunction

Renal dysfunction was defined by increased serum creatinine, acute graft rejection (biopsy proven), chronic allograft nephropathy (biopsy proven), and recurrence of the initial nephropathy. During the follow up phase, 8 patients (16.7%) from the MMF group and 5 patients (22.7%) from the control group experienced at least one renal dysfunction, with 10 patients (17.9%) in group I and 3 patients (21.4%) in group II (Table 3). For the entire duration of the study (5 years), the number of patients with at least one episode of renal dysfunction was 8 in the MMF group, 6 in the control group (27.3%), 10 in group I, and 4 in group II (28.6%).

#### 3.4.3. Safety

Only serious adverse events (SAEs) were recorded during this study. A total of 47 events were reported for 29 patients in the follow up phase. This included 36 SAEs observed in 21 patients (43.8%) of the MMF group and 11 SAEs in 8 patients (36.4%) of the control group. This corresponds to 36 SAEs in 23 patients (41.1%) of group I, and 11 events in six patients (42.9%) in group II (Table 4). No statistical significant difference between the groups was observed.

Most frequently declared SAEs included infections and infestations (11.4% of total patient population, 12.5% versus 9.1% in the MMF group and control group, resp.), cardiac disorders (7.1% of total patient population, 8.3% versus 4.6% in the MMF group and control group, resp.), benign, malignant, or unspecified tumors (7.1% of total patient population, 6.3% versus 9.1% in the MMF group and control group, resp.), and surgical and medical interventions (5.7% of total patient population, 8.3% versus 0% in the MMF group and control group, resp.). Of note, no opportunistic viral infection was reported, and gastrointestinal disorders concerned only three patients.

#### 3.4.4. Laboratory Values and Physical Exams

The evolution of mean uremia, cholesterol, triglycerides, HDL cholesterol, and proteinuria levels during the course of the study was analyzed. No statistically or clinically significant difference was found between MMF versus control group and between group I versus group II at month 60, nor between changes between baseline and month 60 within the populations. Six patients in the MMF group and one patient in the control group had a proteinuria level superior to 3 g/24 h at one or more assessment points during the three-year follow up phase (data not shown). Finally, both mean systolic and diastolic blood pressure (SBP, DBP) varied little during the follow up phase in any of the groups analyzed.

## 4. Discussion

Despite the efficacy of CsA in the prevention of acute graft rejection and improvement of short-term graft survival, CNI-associated nephrotoxicity remains a causal factor to chronic allograft dysfunction and thus limits long-term graft survival [14]. And histological markers of CsA-induced nephrotoxicity as identified by renal graft biopsies are universally present in renal allografts ten years after transplantation [15]. 

The results of the present study concern only CsA. While tacrolimus is also a CNI, his exact impact remains still debated [16]. Some studies confirm the positive impact concerning tacrolimus treatment especially on renal function. An association was demonstrated between the tacrolimus dose and the renal function status. *J. Pascual and al. * highlighted that everolimus with very low tacrolimus regimen had clinically better renal function compared to the low exposure tacrolimus arm [17]. Moreover, in the SYMPHONY study, daclizumab, MMF, and corticosteroids in combination with low-dose tacrolimus demonstrated a better renal function, allograft survival, and acute rejection rates as compared with regimens containing either low-dose CsA or Sirolimus [18]. on the other hand, an increase in interstitial fibrosis and tubular atrophy (IF/TA) was associated with a decrease in kidney function within patients taking tacrolimus, MMF, and prednisone. Indeed, it has repeatedly shown that IF/TA is associated with both allograft dysfunction and allograft loss [19]. Moreover, other factors could also be implicated with tacrolimus nephrotoxicity such as the cytochrome genotype [20, 21]

Hence this demands alternative long-term immunosuppressive treatment options that reduce renal toxicity while maintaining immunosuppression and preventing graft loss. Complete CNI avoidance strategies proved mostly unsatisfactory and resulted in increased acute rejection risk [22–24]. CNI reduction or withdrawal regimens based on nonnephrotoxic immunosuppressive agents such as MMF [25–28] or sirolimus [29–31] yielded promising results regarding improved renal function; however, some of these protocols pose an increased risk for acute rejection, as demonstrated in CsA withdrawal under MMF treatment in de novo transplant patients [25, 26], as well as in late withdrawal in patients with stable renal function [27, 28]. Furthermore, the use of sirolimus is limited due to the high rate of adverse events [32], and results that suggest improved renal function after CsA withdrawal in sirolimus-treated patients [29, 30, 33] may be confounded by an increased risk of toxicity resulting from CsA and sirolimus coadministration in the control groups. Another alternative has recently been introduced. Indeed, Belatacept, a new immunosuppressive therapy, allows avoiding the renal toxicities associated with CNI. Yet, it is associated with a higher incidence and severity of acute rejection episodes than with CsA regimen based [34].

The REFERENCE study was one of the first randomized and controlled studies to show that CsA reduction in the presence of MMF improves renal function in renal transplant recipients with impaired renal function [13]. Yet, the question about long term risks and benefits arising from this treatment remained open. The goal of the present three-year follow up phase was to evaluate the safety of the treatment strategy and the maintenance of the therapeutic response together with improved renal function.

Epidemiologic studies have shown that serum creatinine and changes thereof are predictive for long-term graft survival [35, 36]. Even in the light of conflicting data, serum creatinine is thus considered as useful marker for the survival of renal allografts [14, 37]. In the follow up phase, when results were analyzed according to the initial randomization groups, improvement of renal function as confirmed in the initial study phase by a positive evolution of 1/SeCr and improved creatinine clearance in the MMF group slowly tapered off during the post-trial phase. However, 1/SeCr levels at month 60 remained significantly higher compared to baseline in the MMF group. In addition, patients in the on-treatment MMF group had statistically significant better 1/SeCr levels compared to the on-treatment CsA group at month 60. Furthermore, comparable results were found in the MMF group and in the control group regarding laboratory parameters, including proteinuria and the incidence of SAEs in the course of the three-year follow up phase. This indicates the absence of a detrimental effect of the regimen on the long term. The possibility of an unfavorable long-term effect was raised by the observation that improvement of renal function occurred relatively early during the initial study phase (16 weeks after randomization). This effect might suggest hemodynamic mechanisms (loss of CsA-mediated vasoconstriction [38]) at the origin of the observed improvement, and which might be attenuated by long-term reduced immunosuppression. Such an effect is, however, refuted by the results of the follow up phase. 

Of further interest is the lack of any additional aggravation in the CsA group in the course of the study, which is in contrast to the findings by Dudley et al [39]. This observation could be explained by the design of the present study. After completion of the initial study phase, 50% of the patients in the control group conversed to MMF treatment and had therefore their CsA dose reduced (thus the reduction of the mean daily CsA dose during the follow up phase in that group). The stabilization of renal damage in the control group during the post-trial period might thus be due to reduced CsA doses or MMF specific. Evidence from animal models suggests that MMF may exert a positive effect on renal damage by antifibrotic properties mediated by its antiproliferative action on both immune and nonimmune cells, including renal tubular cells and vascular smooth muscle cells [8]. We sought to eliminate this protocol-related bias by analyzing the perprotocol population, but in this case the number of patients remaining in group II (14 patients) was low for statistical analysis.

A potential risk of CNI-reducing regimens is an increased acute graft rejection rate, as observed in patients with stable renal function [28]. Yet in our study, only a single case of acute rejection was reported throughout the entire post-trial phase, supporting the findings of previous studies in renal function compromised patients [39, 40]. While the absence of protocol biopsies is limiting the interpretation of these results, it remains unlikely that a clinical graft rejection, even if it was initially subclinical, would remain undetected (e.g., no increase of serum creatinine), given the duration of this study. Together with the results from the initial study phase (no biopsy-proven acute rejection, graft survival in the MMF group and control group 99% and 97%, resp.), this suggests that the conversion period (4 weeks of gradual CsA reduction) as well as the study design ensure an adequate tolerance for the therapeutic strategy used. This is corroborated by the low incidence of MMF adverse events, especially of the more frequently reported ones, such as digestive disorders [41].

CsA-reducing regimens were shown to reduce cardiovascular risk factors including hypercholesterolemia and hypertension [39, 40] and may therefore reduce cardiovascular disorders which contribute to mortality and graft loss in renal transplant patients [42–44]. In our study lipid profile and blood pressure were comparable in the MMF group and in the control group, without any clinically significant incidents. Again, this result might be explained by the reduced CsA dose patients received in the control group during the three-year follow up phase.

Furthermore, recent studies demonstrated a statistically significant reduction of tumor rates in CsA-reduced regimens. The only death that occurred during this study and which was the consequence of a neoplastic complication (lung carcinoma) concerned the control group.

The methodological limitations inherent to this type of studies leave the comparison of the two study groups difficult, since a significant number of patients from the control group had their treatment changed to MMF.

Overall, the five-year REFERENCE study shows that the regimen involving CNI reduction in combination with MMF treatment provides a favorable benefit/risk ratio. There was no secondary aggravation related to immunological phenomena, and improvement of allograft function as analysed by 1/SeCr levels was sustained in the long term. Importantly, this study ended on average 12-year post-transplantation for the majority of patients. The published half-life of renal allografts is ca. 8 years for deceased-donor allografts and 12 years for living-donor allografts [45]. Our findings suggest that the study regimen may retard allograft deterioration.

## Figures and Tables

**Table 1:** Study withdrawal and reason for withdrawal.

	Randomization population^1^		On-treatment population^2^		Total
	MMF group^3^ (*n* = 48)	Control group^4^ (*n* = 22)	Group I^5^ (*n* = 56)	Group II^6^ (*n* = 14)	(*n* = 70)
Study withdrawal	5 (10.4%)	3 (13.6%)	7 (12.5%)	1 (7.1%)	8 (11.4%)
Graft loss	2 (4.2%)	2 (9.1%)	3 (5.4%)	1 (7.1%)	4 (5.7%)
*P*-value^7^	.585		1.000		
Death	0 (0%)	1 (4.5%)	1 (4.5%)	0 (0%)	1 (1.4%)
Other^8^	3 (6.3%)	0 (0%)	3 (5.4%)	0 (0%)	3 (4.3%)

^1^Patients randomized to receive either MMF or CsA treatment in the initial study phase.

^2^Determined by the treatment patients received at the end of the post-trial phase (mycophenolic acid derivative or not).

^3^Patients who received 2 g MMF per day and 50% of the initial CsA dose.

^4^Patients who received the usual CsA dose.

^5^Patients who received a treatment with a mycophenolic acid derivative at the end of the follow up phase.

^6^Patients without a mycophenolic acid derivative at the end of the follow up phase.

^7^1 patient moved, 2 patients did not perform visit at month 60.

^8^Comparison between groups of patients who had graft loss.

**Table:** (a) MMF treatment.

	Randomization population^1^				On-treatment population^2^			
	MMF group^3^		Control group^4^		Group I^5^		Group II^6^	
	Patients under MMF 2g/day	Mean MMF dose/day	Patients under MMF treatment	Mean MMF dose/day	Patients under MMF 2 g/day	Mean MMF dose/day	Patients under MMF treatment	Mean MMF dose/day
M30	36 (75.0%)	1844 ± 295 mg	2 (9.0%)	125 ± 448 mg	36 (64.3%)	1549 ± 741 mg	3 (21.4%)	321 ± 668 mg
M48	31 (70.5%)	1773 ± 424 mg	6 (33.4%)	500 ± 786 mg	33 (66.0%)	1650 ± 600 mg	3 (25.0%)	375 ± 711 mg
M60	26 (60.5%)	1628 ± 608 mg	9 (47.4%)	711 ± 822 mg	29 (59.2%)	1704 ± 432 mg	0	—

^1^Patients randomized to receive either MMF or CsA treatment in the initial study phase.

^2^Determined by the treatment patients received at the end of the post-trial phase (mycophenolic acid derivative or not).

^3^Patients who received 2 g MMF per day and 50% of the initial CsA dose.

^4^Patients who received the usual CsA dose

^5^Patients who received a treatment with a mycophenolic acid derivative at the end of the follow up phase.

^6^Patients without a mycophenolic acid derivative at the end of the follow up phase.

**Table:** (b) Mean CsA dose per day.

	Randomization population^1^		On-treatment population^2^	
	MMF group^3^	Control group^4^	Group I^5^	Group II^6^
M30	129 ± 37 mg	190 ± 50 mg	137 ± 48 mg	191 ± 30 mg
M48	134 ± 47 mg	180 ± 54 mg	134 ± 41 mg	205 ± 60 mg
M60	126 ± 44 mg	163 ± 43 mg	128 ± 37 mg	173 ± 63 mg

^1^Patients randomized to receive either MMF or CsA treatment in the initial study phase.

^2^Determined by the treatment patients received at the end of the post-trial phase (mycophenolic acid derivative or not).

^3^Patients who received 2 g MMF per day and 50% of the initial CsA dose.

^4^Patients who received the usual CsA dose.

^5^Patients who received a treatment with a mycophenolic acid derivative at the end of the follow up phase.

^6^Patients without a mycophenolic acid derivative at the end of the follow up phase.

**Table 3:** Renal dysfunction.

	Randomization population^1^		On-treatment population^2^		Total
	MMF group^3^ (*n* = 48)	Control group^4^ (*n* = 22)	Group I^5^ (*n* = 56)	Group II^6^ (*n* = 14)	(*n* = 70)
Number of events	12	6	15	3	18
Increased serum creatinine	7 (58.3%)	2 (33.3%)	8 (53.3%)	1 (33.3%)	9 (50%)
Acute rejection (biopsy proven)	0 (0%)	1 (16.7%)	1 (6.7%)	0 (0%)	1 (5.6%)
Chronic allograft nephropathy (biopsy proven)	3 (25%)	3 (50%)	4 (26.7%)	2 (66.7%)	6 (33.3%)
Recurrence of initial nephropathy	1 (8.3%)	0 (0%)	1 (6.7%)	0 (0%)	1 (5.6%)
Others	1 (8.3%)	0 (0%)	1 (6.7%)	0 (0%)	1 (5.6%)
Patients with at least one episode of renal dysfunction	8 (16.7%)	5 (22.7%)	10 (17.9%)	3 (21.4%)	13 (18.6%)

^1^Patients randomized to receive either MMF or CsA treatment in the initial study phase.

^2^ Determined by the treatment patients received at the end of the post-trial phase (mycophenolic acid derivative or not).

^3^Patients who received 2 g MMF per day and 50% of the initial CsA dose.

^4^Patients who received the usual CsA dose.

^5^Patients who received a treatment with a mycophenolic acid derivative at the end of the follow up phase.

^6^Patients without a mycophenolic acid derivative at the end of the follow up phase.

**Table 4:** Serious Adverse Events (SAEs) during the three-year post-trial phase.

	Randomization population^1^				On-treatment population^2^					
	MMF group^3^(*N* = 48)		Control group^4^(*N* = 22)		Group I^5^(*N* = 56)		Group II^6^(*N* = 14)		Total(*N* = 70)	
	nE	nP	nE	nP	nE	nP	nE	nP	nE	nP
Total SAEs	36		11		36		11		47	

Total patients with at least one SAE		21 (43.8%)		8 (36.4%)		23 (41.1%)		6 (42.9%)		29 (41.4%)

*P*-value				.560				.903		

Infections*	8	6 (12.5%)	2	2 (9.1%)	8	6 (10.7%)	2	2 (14.3%)	10	8 (11.4%)
Cardiac disorders*	4	4 (8.3%)	1	1 (4.6%)	3	3 (5.4%)	2	2 (14.3%)	5	5 (7.1%)
Tumors* (benign, malignant, not specified)	3	3 (6.3%)	2	2 (9.1%)	4	4 (7.1%)	1	1 (7.1%)	5	5 (7.1%)
Surgical and medical interventions*	4	4 (8.3%)	—	—	3	3 (5.4%)	1	1 (7.1%)	4	4 (5.7%)
Gastrointestinal disorders*	2	2 (4.2%)	1	1 (4.6%)	2	2 (3.6%)	1	1 (7.1%)	3	3 (4.3%)
Urinary system and kidney disorders*	2	2 (4.2%)	1	1 (4.6%)	3	3 (5.4%)	—	—	3	3 (4.3%)
Respiratory, thoracic, and mediastinal disorders*	3	3 (6.3%)	—	—	2	2 (3.6%)	1	1 (7.1%)	3	3 (4.3%)

^1^Patients randomized to receive either MMF or CsA treatment in the initial study phase.

^2^Determined by the treatment patients received at the end of the post-trial phase (mycophenolic acid derivative or not).

^3^Patients who received 2 g MMF per day and 50% of the initial CsA dose.

^4^Patients who received the usual CsA dose.

^5^Patients who received a treatment with a mycophenolic acid derivative at the end of the follow up phase.

^6^Patients without a mycophenolic acid derivative at the end of the follow up phase.

Note: percentages were calculated based on the number of patients per group, nE: number of events, nP: number of patients. * details of SAEs per system/organ were done for ones with an incidence ≥3%.
